# Estimating the global burden of Epstein–Barr virus-related cancers

**DOI:** 10.1007/s00432-021-03824-y

**Published:** 2021-10-27

**Authors:** Yide Wong, Michael T. Meehan, Scott R. Burrows, Denise L. Doolan, John J. Miles

**Affiliations:** 1 Australian Institute of Tropical Health and Medicine, James Cook University, Cairns, QLD 4878 Australia; 2 Centre for Molecular Therapeutics, James Cook University, Cairns, QLD 4870 Australia; 3 Centre for Tropical Bioinformatics and Molecular Biology, James Cook University, Cairns, QLD 4878 Australia; 4 Australian Institute of Tropical Health and Medicine, James Cook University, Townsville, QLD 4811 Australia; 5 QIMR Berghofer Medical Research Institute, Herston, QLD 4006 Australia; 6 Faculty of Medicine, The University of Queensland, Herston, QLD 4006 Australia

**Keywords:** Epstein–Barr virus, EBV, Diffuse large B-cell lymphoma, Extranodal NK/T-cell lymphoma, EBV-associated cancer, EBV-related cancer, EBV-related cancer incidence, EBV-related cancer frequency

## Abstract

**Background:**

More than 90% of the adult population globally is chronically infected by the Epstein–Barr virus (EBV). It is well established that EBV is associated with a number of malignancies, and advances in knowledge of EBV-related malignancies are being made every year. Several studies have analysed the global epidemiology and geographic distribution of EBV-related cancers. However, most have only described a single cancer type or subtype in isolation or limited their study to the three or four most common EBV-related cancers. This review will present an overview on the spectrum of cancers linked to EBV based on observations of associations and proportions in the published literature while also using these observations to estimate the incidence and mortality burden of some of these cancers.

**Method:**

We have reviewed the literature on defining features, distribution and outcomes across six cancers with a relatively large EBV-related case burden: Nasopharyngeal carcinoma (NPC), Gastric carcinoma (GC), Hodgkin lymphoma (HL), Burkitt lymphoma (BL), Diffuse large B-cell lymphoma (DLBCL) and Extranodal NK/T-cell lymphoma, Nasal type (ENKTL-NT). We retrieved published region-specific EBV-related case proportions for NPC, GC, HL and BL and performed meta-analyses on pooled region-specific studies of EBV-related case proportions for DLBCL and ENKTL-NT. We match these pooled proportions with their respective regional incidence and mortality numbers retrieved from a publicly available cancer database. Additionally, we also reviewed the literature on several other less common EBV-related cancers to summarize their key characteristics herein.

**Conclusion:**

We estimated that EBV-related cases from these six cancers accounted for 239,700–357,900 new cases and 137,900–208,700 deaths in 2020. This review highlights the significant global impact of EBV-related cancers and extends the spectrum of disease that could benefit from an EBV-specific therapeutic.

**Supplementary Information:**

The online version contains supplementary material available at 10.1007/s00432-021-03824-y.

## Background

Epstein–Barr virus (EBV), also known as Human herpesvirus 4 (HHV4), is a ubiquitous γ-herpesvirus that infects more than 90% of adults globally (Longnecker and Neipel 2007; Dunmire et al. 2018). Primary infections are typically acquired orally during childhood or adolescence, but the age of primary infection has been gradually increasing over time in developed countries as greater socioeconomic status is associated with lower age-specific antibody prevalence (Dunmire et al. 2018).

As part of the herpesvirus family, EBV has a linear double-stranded DNA genome approximately 170 kb in size that circularizes after infecting a host cell and has both a lytic and latent phase of gene expression (Weidner-Glunde et al. 2020). During the lytic phase, a majority of EBV genes are expressed to facilitate the replication of the genome and the production of viral particles (Weidner-Glunde et al. 2020). The latent phase minimizes gene expression while maintaining a persistent infection in the form of episomes that replicate alongside the host chromosomes within a small number of circulating cells (Farrell 2019; Weidner-Glunde et al. 2020). It has not been fully elucidated if the oropharyngeal epithelial cells or naïve B-cells are first to be infected but, eventually, a reservoir of persistent life-long latent infection is maintained in a small number of memory B-cells (Dunmire et al. 2018). These EBV-infected B-cells can then differentiate into plasma cells and reactivate the lytic EBV gene expression profile which allows them to traffic EBV back to the oropharynx for further transmission through saliva (Dunmire et al. 2018; Farrell 2019).

Since its discovery in 1964, EBV was found to be linked with a broad range of cancers throughout the globe (AbuSalah et al. 2020). In 1997, EBV was classified as a group 1 carcinogen by the International Agency for Research on Cancer (IARC) because of its causal association with Endemic Burkitt lymphoma (eBL), Hodgkin lymphoma (HL) and Nasopharyngeal carcinoma (NPC) (IARC 1997). In recent years, EBV was also linked to cancers such as Extranodal NK/T-cell lymphoma, Nasal type (ENKTL-NT), subgroups of Diffuse large B-cell lymphoma (DLBCL) and Gastric Carcinoma (GC), in addition to a number of other non-malignant diseases such as infectious mononucleosis (IM), multiple sclerosis, and oral hairy leukoplakia (AbuSalah et al. 2020).

Several EBV-related cancers exhibit a distinct geography-specific distribution (elaborated elsewhere in this review) and some of these distributions have been attributed to phylogeographical variations between different EBV strains (Jemal et al. 2011; Corvalan et al. 2019). Early attempts to classify these differences led to the discovery of EBV Types 1 and 2 (or A and B) based on differences in genetic sequences (Zimber et al. 1986; Chang et al. 2009). The Type 1 strain is the most common globally; although Type 2 strains represent a smaller proportion of the total global infection burden, they are detected at higher frequencies in Africa, Alaska and Papua New Guinea as compared to Asia, Europe and the Americas (Zimber et al. 1986; Chang et al. 2009). Co-infections with both Type 1 and Type 2 EBV strains are not unusual, especially in areas where EBV Type 2 is more frequent and among immunocompromised individuals (Sculley et al. 1990; Crawford et al. 2006; Neves et al. 2015; Smith et al. 2019). In studies comparing the prevalence of different EBV strains amongst EBV-related cancers, Type 1 was the most common (Gledhill et al. 1991; Suzumiya et al. 1999; Gualco et al. 2011; Neves et al. 2017; Montes-Mojarro et al. 2020a, b). However, one case controlled study on Caucasian patients in Portugal found that EBV Type 2 was more significantly associated with risk of NPC despite the prevalence of EBV Type 1 in the region (Neves et al. 2015). Several geography-specific differences in EBV sequences have been linked with other EBV-associated cancers such as HL and EBV-associated Gastric carcinoma (EBVaGC) (Corvalan et al. 2006; Neves et al. 2017). Most recently, a study exploring the effect of recombination and mutation on EBV phylogenetics has categorized EBV strains into 12 different groups, each with their own geographic and disease association patterns (Zanella et al. 2019).

Other factors have also been found to contribute to the distinct geographical patterns of some EBV-related cancers. Ethnicity and country of birth have been implicated as a risk factor for NPC and these likely relate to lifestyle factors such as diet as well as genetic factors such as the presence of single nucleotide polymorphisms (SNPs) in human leukocyte antigen (HLA) genes (Su et al. 2013; Bakkalci et al. 2020). In Africa, eBL is strongly associated with *Plasmodium falciparum* malaria as evidenced by the overlap in prevalence of *P. falciparum* and eBL (Hammerl et al. 2019; Quintana et al. 2020). Overall, however, only a fraction of individuals infected with EBV develop EBV-related cancers and the risk factors for developing EBV-related cancers are multifactorial and heavily influenced by both environment and genetics. Despite dedicated efforts to understand this phenomenon, the exact causal mechanism responsible for the development of EBV-related cancers in only a proportion of the EBV-infected population has not been fully elucidated and remains a broad target for future research.

Having both immunogenic and oncogenic activity, EBV infection can have significant consequences on the host. No commercial EBV vaccine exists nor do any EBV anti-viral drugs. Treatment of EBV-positive cancers involves classic regimes such as surgery, radiotherapy and/or chemoradiotherapy. Responses to treatments vary by type and stage of cancer. Several cancers exhibit high relapse rates, while others are highly aggressive and resistant to treatment. More recently, immune checkpoint inhibitors have been approved by the FDA to treat metastatic or recurrent locally advanced gastric carcinoma expressing the protein programmed death-ligand 1 (PD-L1) and relapsed or refractory HL (Fashoyin-Aje et al. 2019; Al Hadidi and Lee 2020).

Two large studies that analysed the global burden of some EBV-related cancers were published recently. Referencing the Global Burden of Disease (GBD) study 2017 (Roth et al. 2018), Khan et al. estimated that 265,000 new cases of NPC, HL, BL and GC could be attributed to EBV while a separate study by de Martel et al. referencing the GLOBOCAN 2018 Cancer database estimated that 156,000 new cases of NPC, HL and BL could be attributed to EBV in 2018 (de Martel et al. 2020; Khan et al. 2020). This review will present an overview on the spectrum of cancers linked to EBV based on observations of associations and EBV-related case proportions in the published literature while also using these observations to estimate the incidence and mortality burden of some of these cancers. This review is not limited to only cancers that have been causally associated with EBV but also cancers that are considered EBV-related yet still lacks strong evidence for the virus’ causal relationship. This review seeks to expand and supplement the studies above by estimating the EBV-related incident and mortality burden of six cancers with large EBV-related case numbers: NPC, HL, GC, BL, DLBCL and ENKTL-NT. A publicly available cancer database from 2020 with cancer incidence and mortality numbers will be combined with EBV-related case proportions and cancer subtype frequencies extracted from the literature, factoring in regional differences where possible and performing meta-analyses when required. Additionally, this review describes a number of other less common EBV-related cancers as defined above and highlights the possible significance of a cancer therapy specifically directed against EBV.

## Methods

Cancer incidence and mortality numbers were extracted directly from GLOBOCAN project via the Cancer Today website compiled and released by the World Health Organization International Agency for Research on Cancer (IARC) (Ferlay et al. 2020; Sung et al. 2021). These mortality and incidence estimates for 2020 were broken down into 36 major cancer types across 185 countries. The database also aggregated countries into pre-defined geographical groupings such as World Health Organization (WHO) regions, continents or world areas. Cancer numbers for 2020 were presented as point estimates with a range of incidences or mortality per geographical area.

### Estimating the incidence and mortality of EBV-related nasopharyngeal carcinoma, Hodgkin lymphoma and gastric carcinoma

NPC, HL and GC were classified under individual cancer entries in the Cancer Today website. The frequencies and incidence of NPC and HL cases causally associated with EBV was previously analyzed and published (Plummer et al. 2016; de Martel et al. 2020). The association frequencies with 95% confidence intervals of HL for the respective continents was directly extracted for calculations in this review from the 2016 study by Plummer et al. (2016). For NPC, no confidence interval or defined geographical breakdown was published in these studies thus the global EBV-associated case proportion used in this review was calculated by working out the proportion of new NPC cases attributable to EBV of all new NPC cases, which were both described in the 2020 study by de Martel et al. (2020). For GC, the EBV-related case frequencies with 95% confidence intervals were extracted from two published meta-analyses that pooled studies from the Americas (Carrasco-Avino et al. 2017) or global studies (Tavakoli et al. 2020).

The lower range of EBV-related cancer incidence estimates for the respective regions were calculated as the lower published 95% confidence interval of EBV-related case proportions for the respective cancer in the respective geographical area multiplied by the lower uncertainty interval of its incidence from Cancer Today. The upper estimate of EBV-related incidence was calculated similarly using the upper confidence intervals of EBV-related case proportions and incidence numbers (Supplementary Table 1). For NPC, due to the reasons described above, a single global EBV-related case proportion was used instead. The upper and lower mortality ranges of EBV-related cancers were extrapolated in the same manner to the incidence figures from the Cancer Today website (Supplementary Table 2).

### Estimating the incidence and mortality of EBV-related diffuse large B-cell lymphoma, extranodal NK/T-cell lymphoma and Burkitt lymphoma

DLBCL, ENKTL-NT and Burkitt Lymphoma (BL) do not have individual entries in the Cancer Today website and are classified under the broad category of Non-Hodgkin Lymphomas (NHL). To estimate the incidences for DLBCL and ENKTL-NT and proportions of EBV-related cases, we conducted several meta-analyses of proportions with a random effects model using the meta-package in the R programming language (version 4.0.2) (Balduzzi et al. 2019). Studies describing the frequency of DLBCL as a proportion of NHL (Chang et al. 2005; Aoki et al. 2008; Chen et al. 2008; Okan et al. 2008; Engels et al. 2010a, b; Yoon et al. 2010; Kim et al. 2011; Wada et al. 2011; Yang et al. 2011; Laurini et al. 2012; Sun et al. 2012; Szumera-Cieckiewicz et al. 2014; van Leeuwen et al. 2014; Al-Hamadani et al. 2015; Cao et al. 2018; Intragumtornchai et al. 2018), EBV-related DLBCL as a proportion of DLBCL (Hoeller et al. 2010; Hofscheier et al. 2011; Wada et al. 2011; Ozsan et al. 2013; Pan et al. 2013; Ok et al. 2014; Sato et al. 2014; Xie et al. 2014; Hong et al. 2015; Lu et al. 2015; Monabati et al. 2016; Stuhlmann-Laeisz et al. 2016; Cohen et al. 2017; Ohashi et al. 2017; Okamoto et al. 2017; Tokuyama et al. 2017; Beltran et al. 2018; Miyagi et al. 2020) and ENKTL-NT as a proportion of NHL (Aoki et al. 2008; Yoon et al. 2010; Kim et al. 2011; Yang et al. 2011; Laurini et al. 2012; Sun et al. 2012; Szumera-Cieckiewicz et al. 2014; van Leeuwen et al. 2014; Aviles 2015; Cao et al. 2018; Intragumtornchai et al. 2018; Agrawal et al. 2021) were retrieved from the published literature. Through this analysis, 95% confidence intervals for each individual study were calculated along with an estimate for the pooled proportion and its 95% confidence interval. Heterogeneity was assessed using Higgins *I*^2^ statistics (Higgins et al. 2003), which revealed significant heterogeneity between studies in all cases (Supplementary Figs. 1, 2, 3). The calculated 95% confidence intervals of both EBV-related case proportion and frequency of all NHL cases for these lymphomas in the respective regions were multiplied by the upper and lower uncertainty intervals of NHL incidence and mortality numbers for the respective lymphoma and region from Cancer Today (Supplementary Table 3). The frequencies and incidence of BL cases attributable to EBV in 2018 was also previously analyzed in the 2020 study by de Martel et al. (2020). However, no confidence interval was published and the geographical regions were not well defined. Similar to NPC mentioned previously, the global EBV-associated case proportion out of all BL cases and NHL cases was estimated by calculating the proportion of new BL cases attributable to EBV against all new BL cases as described by de Martel et al. and the central estimate of all new NHL cases in 2020 from the Cancer Today website, respectively. The EBV-related cancer mortality range for BL was estimated by multiplying the EBV-associated case proportion out of all NHL cases by the upper and lower mortality numbers from the Cancer Today website (Supplementary Table 2).

## Global cancer impact of common EBV-related cancers

### Nasopharyngeal carcinoma

NPC arises in the lateral walls of the nasopharynx and is characterized by a rich submucosal lymphatic network leading to early development of cervical lymph node metastasis. Given the anatomic location, NPC is usually considered unrespectable. Primary treatment is radiotherapy or chemoradiotherapy, and around a third of patients present with locoregional recurrence and distant metastases (Lee et al. 2005; Le et al. 2019). Relapse patients are only amenable to palliative radiotherapy or chemotherapy and overall median survival is between 7 and 22 months (Ma and Chan 2006; Caponigro et al. 2010; Bensouda et al. 2011). In 2020, there were an estimated 124,700–142,500 incident cases of NPC and 72,800–87,800 deaths globally (Ferlay et al. 2020; Sung et al. 2021). In areas with high incidence of NPC such as Eastern, South-Eastern Asia and some areas in the Middle-East, EBV is associated with more than 95% of NPC cases. However, in other areas of low NPC incidence, NPC cases are comparatively fewer and the EBV association rates could be as low 75% (Yu and Yuan 2002; Peterson and Nelson 2013). Cumulatively, it was estimated that approximately 84.6% of all new NPC cases could be attributed to EBV (de Martel et al. 2020). NPC has a distinctive genetic distribution in individuals from Asia (South-East Asia and North-East India), North Africa, Northern Canada, Alaska and the Pacific. The incidence of NPC is high in Singapore, Malaysia, the Philippines, Vietnam and Indonesia. Overall, in South-East Asia, NPC is the sixth most common cancer in males (Jemal et al. 2011; Adham et al. 2012). Among the Chinese of Taiwan, NPC is the most common cancer in males and the third most common cancer in females (Hsu et al. 1982). In Hong Kong and regions of Southern China, the annual incidence rates reach 50 per 100,000 individuals (Yu and Yuan 2002; Smith and Khanna 2012). Overall, NPC is reported to be the eighth leading cause of cancer death in China and a leading cause of cancer death in the Hong Kong, Guangdong and Guangxi populations (Guo et al. 2009). There are increasing incidences in Australia and the United States through increased immigration from Asia, emphasizing the growing importance of EBV-related cancers on the global scale (Jemal et al. 2011).

### Gastric carcinoma

GC accounted for approximately 748,600–789,500 deaths and 1,066,500–1,112,100 new cases of cancer worldwide in 2020 (Ferlay et al. 2020; Sung et al. 2021). Despite the overall decline in incidence and mortality in developed countries, GC still has the fifth highest incidence and fourth highest mortality among cancers worldwide (Catalano et al. 2009). Historically, categorization of GC cases was based on histology and anatomical location. Recently, the Cancer Genome Atlas Research Network (TCGA) found that GC cases can be broken down into four distinct classifications, each with its own molecular signature and clinical characteristics (Cancer Genome Atlas Research 2014). Microsatellite Instability (MSI) GC cases are characterized by higher mutation rates and hypermethylation at the MutL homolog 1 (MLH1) promoter. Genomically Stable (GS) GC cases are characterized by mutations and/or fusions of proteins associated with the Rho family of GTPases. Chromosomal Instability (CIN) GC cases are characterized by higher levels of somatic copy number aberrations. The fourth group, EBVaGC, is the only EBV-related subtype and cases are characterized by the presence of EBV in the cancer cells together with CDKN2A promoter hypermethylation. Of note, EBVaGC cases have the most frequent DNA hypermethylation overall compared to the other three groups of GC (Cancer Genome Atlas Research 2014; Rodriquenz et al. 2020). Described as an EBV-specific methylation epigenotype, more than 800 genes expressed by the EBV-infected AGS-EBV GC cell line were found to have CPG hypermethylation, and many of these genes were associated with the development of cancer (Kaneda et al. 2012; Zhao et al. 2013). The causal association with EBV is still inconclusive due to limitations in existing data. However, the unique molecular profile as mentioned above, as well as the marked differences in Epstein–Barr virus-encoded RNA (EBER) in situ hybridization (ISH) staining of GC cases compared to adjacent non-cancer tissues or peptic ulcer disease controls as well as the presence of monoclonal EBV DNA in EBVaGC tissue supports the possibility of a mechanistic relationship (Imai et al. 1994; Chen et al. 2015). Nonetheless, EBV infection was shown to increase the risk of GC by more than 18 times and its unique molecular profile together with the specific expression of EBER in cancer tissues suggests that this sub-group of GC may benefit from a treatment that targets the virus or its associated oncogenic pathways (Tavakoli et al. 2020).

The frequency of EBVaGC of all GC cases in the Americas is estimated to be 11.49% (95% CI 8.46–15.43%) and 8.7% for the rest of the world (95% CI 7.73–9.92%) (Carrasco-Avino et al. 2017; Tavakoli et al. 2020). Data from another study that followed 4599 GC patients from Asia, Europe and Latin America showed that EBVaGC cases had better prognosis with a median survival of 8.5 years compared to EBV-negative GC which had a median survival of 5.3 years (Camargo et al. 2014), emphasizing the importance of understanding the causal relationship between EBV and GC. There is currently no consensus on the recommended treatment approach for GC. Operable GC including EBVaGC is usually treated with a complete resection and lymphadenectomy that may be supplemented with chemotherapy or radiotherapy (Chon et al. 2017; Sitarz et al. 2018). Advanced or metastatic GC is typically managed palliatively with gastrectomy, radiotherapy or chemotherapy (Chon et al. 2017; Sitarz et al. 2018). More recently, antibodies which block the negative immune regulatory programmed cell death protein 1 (PD-1), pembrolizumab, was approved by the FDA to treat patients with metastatic or unresectable MSI tumours (including MSI GC) as well as patients with metastatic, recurrent or locally advanced GC that expresses PD-L1 and have progressed despite two or more systemic treatments (Fashoyin-Aje et al. 2019). Blocking PD-1 or PD-L1 may also be a promising treatment for EBVaGC specifically as tumours from these cases were more likely to overexpress PD-L1 (Gu et al. 2017). There is as yet no data on the efficacy of PD-1 or PD-L1 blockade as a treatment for EBVaGC but trials are currently being conducted to investigate this approach (Naseem et al. 2018).

### Hodgkin lymphoma

HL is a cancer of B-cell origin which encompasses five different histological subtypes: lymphocyte-rich; lymphocyte-depleted; nodular sclerosis, and mixed cellularity subtypes, which can be collectively labelled as classical HL; as well as the nodular lymphocyte-predominant HL (NLPHL) subtype (Kuppers et al. 2012). The malignant tumour cells are known as Hodgkin and Reed–Sternberg (HRS) cells in classical HL, and lymphocyte-predominant (LP) cells in NLPHL. A defining trait of HL is that these malignant cells make up only 0.1–2% of cells in the tumour (Kuppers et al. 2012). There were 78,800–87,600 new cases of HL and 20,100–27,000 resulting deaths in 2020 (Ferlay et al. 2020; Sung et al. 2021). The fraction of HL attributable to EBV was estimated to be 74% (95% CI 65–82%) in Africa, 60% (95% CI 54–67%) in Latin America, 56% (95% CI 52–60%) in Asia, 36% (95% CI 32–39%) in Europe, 32% (95% CI 25–39%) in North America and 29% (95% CI 10–58%) in Oceania (Plummer et al. 2016; de Martel et al. 2020). Overall survival of patients with early-stage HL disease at diagnosis is more than 90% and 60–80% for advanced-stage disease (Townsend and Linch 2012; Shanbhag and Ambinder 2018). These high overall survival outcomes, achieved through intensive dose chemoradiotherapy and/or chemotherapy regimens, are not without consequence as HL survivors who have received radiation have lower rates of average per-person life expectancy when compared with healthy age-matched controls (Gandhi et al. 2004; Yeh and Diller 2012). Anti-PD-1 antibodies, nivolumab and pembrolizumab have also recently been approved by the FDA for treating relapsed or refractory HLs due to promising results across multiple clinical trials demonstrating an overall response rate of more than 65% (Hu et al. 2018; Al Hadidi and Lee 2020).

### Non-Hodgkin lymphoma

NHLs are a large diverse group of neoplasms that originate from lymphoid cells. NHLs account for more than 75% of all lymphomas, with HL accounting for the other 10–25%. In 2020, NHLs were responsible for more than 254,400–265,200 deaths and 536,000–552,800 new cases of lymphoma (Ferlay et al. 2020; Sung et al. 2021). According to the 2016/17 revision of the WHO classifications of tumours of haematopoietic and lymphoid tissues, there are more than 60 different defined subtypes of NHL (Swerdlow et al. 2016; Campo et al. 2017). The following sections will address the common NHL subtypes that have been linked to EBV.

#### Burkitt lymphoma

BL was first described by Dennis Burkitt in 1958 as a sarcoma afflicting the jaws of African children (Burkitt 1958). It was in cultures of these tumour cells that EBV was discovered by electron microscopy (Epstein et al. 1964). According to the WHO, three groups of BL exist: eBL, which overlaps with *P. falciparum* endemic areas; sporadic BL, which develops outside of *P. falciparum* endemic areas; and immunodeficiency-associated BL (further described below under “EBV-associated Immunosuppressive cancers”) (Molyneux et al. 2012; Chabay et al. 2020). eBL is a prevalent childhood cancer in Equatorial Africa and Papua New Guinea and affects approximately 3–6 per 100,000 children under the age of 14, and between 81 and 100% of all cases are EBV-positive (Magrath 2012; Plummer et al. 2016; Hammerl et al. 2019). In Latin America, EBV association frequencies are lower, occurring in 29–87% of BL cases with frequencies increasing with geographic latitude (Chabay et al. 2020). Approximately 10–15% of sporadic BL cases are EBV-associated and a recent study referencing the GLOBOCAN 2018 database estimated that globally, around 6600 (55%) new cases of BL in 2018 were EBV-associated (Mawson and Majumdar 2017; de Martel et al. 2020). A translocation of genetic information between the C-MYC gene at chromosome 8 and the immunoglobulin heavy chain (IGH) at chromosome 14 is present in approximately 70–80% of BL cases (Molyneux et al. 2012). The geographic regions with high risk of malaria infections and eBL appear to overlap (Hammerl et al. 2019; Quintana et al. 2020). It has been demonstrated that the *P. falciparum* parasite may be inducing MYC translocations via the toll-like receptor 9 and the activation-induced cytidine deaminase in B-cells (Torgbor et al. 2014). In addition, malaria causes immune perturbations that may result in the increased proliferation of EBV-infected B-cells (Yone et al. 2006; Molyneux et al. 2012). In low-income countries, treatment of eBL with low cost chemotherapy may result in 1-year survival rates of 50% (Hesseling et al. 2009). In high-income countries, intensive chemotherapy results in 5-year survival rates of more than 80% (Chantada et al. 1997).

#### Diffuse large B-cell lymphoma

DLBCL is one of the highest frequency groups of NHLs, meta-analyses conducted in this review found that the pooled proportion of DLBCL among cases of NHL was 42.1% (95% CI 35.5–48.9%) in East and South-East Asia, 39.9% (95% CI 30–50.7%) in Latin America and 25.6% (95% CI 20.9–31%) for the rest of the world (Supplementary Fig. 1). This category of lymphomas are characterised by fast replicating large cells with vesicular nuclei that are usually more than twice the size of small lymphocytes (Martelli et al. 2013). To date, the cause of DLBCL remains unknown in most cases. Risk factors include but are not limited to ionizing radiation, immune suppression and chemicals such as hair dyes, fertilizers and pesticides (Friedberg and Fisher 2008; Martelli et al. 2013). DLBCL is typically treated with R-CHOP (Rituximab, Cyclophosphamide, Doxorubicin, Vincristine and Prednisone) combined with radiotherapy. The 5-year survival with this treatment is 80–85% in early or intermediate stage disease. However, in patients who present with advanced disease or lack access to Rituximab, 5-year survival drops to approximately 50% (Friedberg and Fisher 2008; Martelli et al. 2013; Hedstrom et al. 2014). “EBV-positive DLBCL of the elderly” was recognized as a subtype of DLBCL in the 2008 update of the WHO classification of lymphoid neoplasms as it was thought to predominantly affect the elderly (Swerdlow et al. 2016). However, in the 2016/2017 update of the WHO classification, the term “EBV-positive DLBCL of the elderly” was replaced with “EBV-positive DLBCL, not otherwise specified” (NOS) due to consensus that EBV-positive DLBCL can affect younger patients with both older and younger patients experiencing similarly poor outcomes compared to EBV-negative DLBCL patients (Lu et al. 2015; Swerdlow et al. 2016; Campo et al. 2017). EBV-related DLBCL case proportion is typically determined through Epstein–Barr virus-encoded RNA (EBER) in situ hybridization (ISH) of the tumour sample to quantify the proportion of EBER-positive tumour cells. There has yet to be a consensus on the threshold required to classify an EBV-positive case since the threshold rate used in previous studies varied between 10 and 50% EBER-positive cells (Castillo et al. 2018). Given that studies have observed significantly different outcomes between EBV-positive and EBV-negative DLBCL patients classified according to a 20% EBER positivity threshold, subsequent meta-analyses in this section will consider a case EBV-positive if the sample contains a minimum of 20% EBER-positive cells (Park et al. 2007; Lu et al. 2015; Hong et al. 2017). Meta-analyses conducted in this review estimated that the pooled proportion of EBV-positive DLBCL among cases classified as DLBCL was 7.2% (95% CI 4.9–10.6%) in East and South-East Asia, 15.2% (95% CI 3–51%) in Latin America and 4% (95% CI 2.4–6.7%) for the rest of the world (Supplementary Fig. 2). Results from the estimates in this review were slightly different but comparable to a previously published meta-analysis which estimated that the pooled proportion of EBV-positive DLBCL was 9.2% (95% CI 7–12%) for Asian and South American populations and 4.7% (95% CI 3.2–6.87%) for Western populations (Hwang et al. 2021). Some of these differences can be explained by the decision to only include studies using the EBV-positive threshold of 20% instead of 10% or more of tumour cells staining for EBER in this review and the different pooling approach used by Hwang et al.

#### Extranodal NK/T-cell lymphoma, nasal type

ENKTL-NT is a relatively uncommon cancer that falls under the sub-group of NHLs known as Mature T and NK neoplasms in the 2016/17 revision of the WHO classification of tumours of haematopoietic and lymphoid tissues (Swerdlow et al. 2016; Campo et al. 2017). Although there has yet to be a consensus on its causal relationship with EBV, EBV genomes are usually clonal in ENKTL-NT cells and by definition, this disease is EBV-related and positive EBER staining is considered one of its key diagnostic markers (Haverkos et al. 2016; Hue et al. 2020). As its name suggests, ENKTL-NT rarely involves the lymph nodes and typically affects the nasal and oral cavity but some patients can present with disease involving other sites such as the skin, digestive tract, lungs or liver (Fox et al. 2020). Common symptoms include facial swelling, bloody or purulent nasal discharge, fever, weight loss and necrotic lesions at the affected site (Kaur et al. 2019; Hue et al. 2020). Meta-analyses conducted in this review estimate that the pooled proportion of ENKTL-NT among cases of NHL was 6.5% (95% CI 3–13.6%) in East and South-East Asia, 2.9% (95% CI 0.9–8.7%) in Latin America and 0.2% (95% CI 0.03–2.1%) for the rest of the world (Supplementary Fig. 3). ENKTL-NT is typically treated with chemotherapy such as the SMILE protocol (dexamethasone, methotrexate, ifosfamide, l-asparaginase and etoposide) alone. However, the addition of radiotherapy to chemotherapy significantly improves outcomes with 5-year overall survival rate of 58% compared to 24% in patients receiving chemotherapy alone (Fox et al. 2020). Of note, patients presenting with nasal ENKTL-NT experienced better outcomes, with a 5-year progression-free survival (PFS) rate of 47% compared to 26% among patients with extranasal ENKTL-NT (Fox et al. 2020). Furthermore, elevated plasma EBV DNA at presentation correlates with adverse clinical grading, while an undetectable EBV DNA load post-SMILE treatment suggests tumour chemosensitivity and predicts for superior survival outcomes (Kwong et al. 2014).

#### Other EBV-related cancers

A number of other cancers have been recognized as EBV-related over the years. However, the incidences of these cancers are comparatively rare. This review has not incorporated these cancers into the estimates of the global burden of EBV-related cancers, but some key features of some of these cancers are discussed below.

## Other EBV-related cancers in immunocompetent individuals

### EBV-Positive Nodal T-cell and NK-cell lymphoma

EBV-positive nodal T- and NK-cell lymphoma is an uncommon form of NHL that has been included as a provisional entity under the sub-group of NHLs known as peripheral T-cell lymphoma, not otherwise specified (PTCL, NOS) in the 2016/2017 revision of the WHO classification of tumours of haematopoietic and lymphoid tissues (Swerdlow et al. 2016; Campo et al. 2017). This disease mainly affects the lymph nodes with patients presenting with lymphadenopathy with limited extranodal involvement (Montes-Mojarro et al. 2020a, b). The presence of EBER-positive cells within the tumour is a requirement to differentiate the cancer from other forms of PTCL, NOS and it is common for more than 50% of neoplastic cells to stain positive (Jeon et al. 2015; Kato et al. 2015). Primary EBV-positive nodal T- and NK-cell lymphoma is primarily treated with chemotherapy such as CHOP but prognosis is poor with a median survival of less than 4 months (Jeon et al. 2015; Kato et al. 2015).

### EBV-positive T-cell and NK-cell lymphoproliferative disease of childhood

In the 2016/2017 revision of the WHO classification of tumours of haematopoietic and lymphoid tissues, the sub-group of NHLs known as EBV-positive T-cell and NK-cell lymphoproliferative disease of childhood encompasses four rare cancers: Systemic EBV-positive T-cell lymphoma of childhood (STCLC); Chronic active EBV infection (CAEBV) of T or NK-cell type, systemic form; Hydroa vacciniforme (HV)-like lymphoproliferative disorder (LPD); and Severe mosquito bite allergy (SMBA). This group of diseases has been reported to more frequently affect children and adolescents from Asia and Indigenous populations of Mexico, Central and South America, and are EBV-associated in more than 90% of cases (Swerdlow et al. 2016; Campo et al. 2017; Kimura and Fujiwara 2018).

Primary EBV infection is typically asymptomatic. In some cases, particularly among those aged 16–20, the infected individual develops IM which manifests itself through symptoms such as sore throat, lymphadenopathy, fatigue and palatine petechiae (Dunmire et al. 2018). IM is also characterized by its long disease course, which can last as long as 12 months (Cameron et al. 2006). The systemic form, CAEBV of T or NK-cell type, is diagnosed when immunocompetent individuals fail to control the initial EBV infection and continue to experience intermittent or persistent IM-like symptoms for more than 3 months, as well as exhibit elevated blood EBV loads and organ involvement with the presence of EBV RNA or protein in the affected tissues (Montes-Mojarro et al. 2020a, b). This disease has the potential to transition into more aggressive lymphomas such as ENKTL-NT or STCLC (Chen and Guan 2019).

HV-like lymphoproliferative disorder (LPD) and SMBA are both cutaneous forms of CAEBV. HV-like LPD causes the formation of papulovesicular lesions, which gradually develop into blisters with vacciniform scarring on areas with regular sun exposure such as the face and back of hands. Cases exist along a spectrum with milder cases resolving themselves after photoprotection and more severe disease potentially developing into lymphomas such as ENKTL-NT or STCLC (Kimura and Fujiwara 2018; Hue et al. 2020; Montes-Mojarro et al. 2020a, b). SMBA is a hypersensitivity reaction characterized by the formation of local ulcers and bullae within hours of a mosquito bite. During disease, patients also experience elevated levels of IgE, NK-cells and EBV load. Patients do not display symptoms once the condition is resolved until another mosquito bite occurs. Patients with a known history of SMBA typically have a higher risk of developing other EBV-associated cancers (Kimura and Fujiwara 2018; Hue et al. 2020; Montes-Mojarro et al. 2020a, b).

STCLC is the most aggressive disease from this sub-group of NHLs and it can occur during the course of CAEBV of T or NK-cell type or after primary EBV infection. Patients present with rapidly developing high fever, hepatosplenomegaly, low white blood cell count, low red blood cell count, low platelet count, abnormal liver function and coagulopathy (Dojcinov et al. 2018; Hue et al. 2020; Montes-Mojarro et al. 2020a, b). Uniquely, anti-VCA IgM antibodies will be low or absent which is unexpected at this stage of EBV infection. Prognosis is poor as most patients die due to complications within days to weeks after diagnosis however, there have been a few cases that responded favourably to chemotherapy with or without stem cell transplantation (Dojcinov et al. 2018; Yoshida et al. 2018).

## Other EBV-related cancers in immunocompromised individuals

### EBV-positive mucocutaneous ulcer

EBV-positive mucocutaneous ulcer (EBVMCU) is a new provisional entity under the sub-group of Mature T and NK neoplasms in the 2016/17 revision of the WHO classification of tumours of haematopoietic and lymphoid tissues (Swerdlow et al. 2016; Campo et al. 2017). EBVMCU was first described in 2010 in a group of patients experiencing immune suppression due to old age or other medical reasons such as receiving immune suppressive treatment for autoimmune disease (Dojcinov et al. 2010). Patients typically present with local lesions on the skin or mucosal surfaces and cases can be classified as skin and oral tumours or oropharyngeal, rectal and genital ulcers (Hess et al. 2020). EBVMCU is thought to be caused by local EBV-associated lymphoproliferation with no evidence of systemic involvement (Daroontum et al. 2018). Some cases are known to self-limit but, for those that do not, strategies including reduction in immunosuppression, chemotherapy, radiation therapy, surgical resection or a combination of the above have demonstrated very favourable responses (Roberts et al. 2015).

### EBV-associated lymphomas in HIV-positive individuals

It is estimated that there were approximately 37,900,000 people living with human immunodeficiency virus (HIV) infections worldwide at the end of 2018 (WHO 2018). While new HIV infection rates have dropped, the availability and usage of antiretroviral therapies have increased. The raised life expectancy of HIV patients has resulted in an increase in individuals living with HIV globally (Fettig et al. 2014). EBV-associated lymphomas are usually caused by the suppression of the immune system by HIV infections which then permits the proliferation of EBV-infected lymphocytes (Carbone et al. 2009a, b).

#### EBV-associated Hodgkin lymphoma in HIV-positive individuals

The risk of developing HL is 5–14 fold higher in HIV infected individuals compared to HIV-negative individuals (Reid et al. 2018). Furthermore, HIV-associated HL tumours are generally more aggressive than HL cases in immunocompetent patients, and the proportion of HRS cells is much higher in comparison (Carbone et al. 2009a, b; Jacobson and Abramson 2012). Treatment is two-pronged, with the aim to control tumour growth with combination chemotherapy and improve immune competence with antiretroviral treatments. Patients with HIV-associated HL typically present with worse prognostic indicators and risk factors compared to their immunocompetent counterparts (Montoto et al. 2012; Besson et al. 2015). Despite this, treatment with antiretroviral therapy and chemotherapy such as adriamycin, bleomycin, vinblastine and dacarbazine (ABVD) has led to comparable outcomes with one study finding a 2-year PFS of 86% for HIV-negative patients and 89% for HIV-positive patients. A separate study found 5-year overall survival rates of 83% for HIV-negative patients and 85% for HIV-positive patients and an identical 5-year event-free survival rate of 64% (Montoto et al. 2012). Unsurprisingly, the EBV-positive frequency of HIV-associated HL is much higher compared to immunocompetent individuals, and ranges from 80 to 100% of all cases (Carbone et al. 2009a, b; Reid et al. 2018).

#### EBV-associated non-Hodgkin lymphoma in HIV-positive individuals

HIV-positive individuals have a tenfold higher risk of developing NHLs compared with HIV-negative individuals (Robbins et al. 2014). HIV-positive NHL patients also present with higher grade disease and with frequent extranodal involvement (Fisher and Fisher 2004). HIV-associated DLBCL is one of the most frequently occurring subtypes of HIV-associated NHL, accounting for approximately 50% of HIV-associated NHL (Linke-Serinsoz et al. 2017; Hernandez-Ramirez et al. 2019). EBV involvement is also more common. Depending on the lymphoma site, 20–90% of HIV-positive DLBCL cases are linked to EBV compared to 4–13% described earlier in this review (Aboulafia et al. 2004; Chao et al. 2012; Linke-Serinsoz et al. 2017).

HIV-associated BL is another frequent subset of HIV-associated NHL, comprising approximately 9–40% of cases (Gibson et al. 2014; Linke-Serinsoz et al. 2017; Hernandez-Ramirez et al. 2019). It is well documented that a drop in CD4 T-cell count increases the risk of NHL dramatically, but unlike most other HIV-associated lymphomas, HIV-associated BL risk is higher for patients with a relatively high CD4 T-count. It was proposed that the BL defining c-MYC translocation is highly apoptotic and as a germinal centre lymphoma, B-cells carrying the c-MYC translocations would require CD4 T-cells to rescue them from apoptosis (Engels et al. 2010a, b; Guech-Ongey et al. 2010; Rodrigo et al. 2012). The result of this reversed interaction with CD4 T-cell numbers is that, unlike HIV-associated DLBCL and other HIV-associated NHLs, the incidence of HIV-associated BL has not decreased in response to the benefits of Highly Active Antiretroviral Therapy (HAART) (Vockerodt et al. 2015). Only 30–40% of HIV-associated BL cases are EBV-positive, which suggests that the pathogenic role of EBV may not be as considerable against the background of HIV induced B-cell dysregulation (Young and Rickinson 2004; Molyneux et al. 2012; Linke-Serinsoz et al. 2017).

HIV-associated NHL cases are typically treated with chemotherapies such as R-CHOP for HIV-associated DLBCL or for HIV-associated BL, the B-ALL/NHL protocol, a regimen adapted from the German Multicentre Study Group on Adult Acute Lymphoblastic Leukaemia (Schommers et al. 2015; Meister et al. 2018). Survival in these patients has improved significantly since the advent of HAART with multiple studies showing 2-year overall survival rates exceeding 60% for both HIV-associated BL and DLBCL (Schommers et al. 2015; Meister et al. 2018; Re et al. 2019). While overall survival may be slightly lower for HIV-positive patients, with combination therapy treatment, outcomes are expected to be largely comparable with HIV-negative patients as HAART reduces the risk of secondary infections. When the treatments are to be used in tandem, toxicity should be taken into account to determine the optimum intensity of either regimen (Lee et al. 2010; Spina et al. 2010).

### Limitations

Studies from different global regions were included herein to increase the resolution of our calculations and to account for geographical differences in EBV-related case proportions. However, some locations were inevitably left out due to the lack of available data, although this may become available with time. Using the uncertainty interval available from published literature, the GLOBOCAN database and meta-analyses may have exaggerated the range of EBV-related cancers. Nonetheless, the approach undertaken in this review is broadly supported by the strategy taken by de Martel et al. and Khan et al. with the exception that this review went one step further by including the uncertainty range of the collected data (de Martel et al. 2020; Khan et al. 2020).

## Conclusions

This review presents an estimate of the extent of EBV involvement in the global burden of cancer. Overall, it is estimated that EBV was linked to approximately 239,700–357,900 new cases of cancer and 137,900–208,700 cancer deaths worldwide in 2020, and approximately 1.3–1.9% of the global cancer burden (Table 1). This incidence is likely to be a conservative estimate as many other rare EBV-related cancers were beyond the scope of this review or remain under-reported and not included in the estimated number. It is projected that these relatively infrequent EBV-related cancers will add approximately 5000–15,000 cases to the annual global burden. The incidence estimate in this review is higher than the 156,000 point estimate calculated by de Martel et al. as this review added EBV-related GC, DLBCL and ENKTL-NT cases which we estimate contribute a further 93,300–178,200 to that number (de Martel et al. 2020). The 265,000 incident cases estimated by Khan et al. did not include EBV-related DLBCL and ENKTL-NT; if these subtypes were to be included, the resulting numbers would likely fall in the middle of our estimated range (Khan et al. 2020). This review has also highlighted that the number of EBV-related cancer classifications are being increasingly recognized, and it is estimated that the total annual global incidence is likely to exceed a quarter of a million. The presence of EBV within these cancers presents a potential therapeutic target for the novel cancer drugs or adoptive T-cell therapy as the virus is a non-self-antigen that might be easier to target. The availability of such therapies could benefit hundreds of thousands worldwide some of whom are suffering extremely aggressive and treatment resistant cancers.

**Table 1:** Estimated global incidence and mortality of EBV-associated cancers in 2020

Cancer type	% estimated global EBV-related case proportion	Estimated incidence range of EBV-related cases	Estimated mortality range of EBV-related cases
NPC	84.6^1^	105,500–120,600	61,600–74,300
GC	7.7–10.4^2^	82,800–116,400	58,200–82,300
HL	45.8–58.3^2^	34,300–52,400	9400–17,400
BL	55^1^	6600^1^	3000–3200
DLBCL	3.6–12.8^2^	4900–27,000	2500–13,300
ENKTL-NT	100^3^	5500–34,700	3000–18,100
Cancer types combined	1.3–1.9^2^	239,700–357,900	137,900–208,700

Published EBV-related case proportions or results from meta-analyses for each cancer were applied to the global cancer incidence and mortality database, Cancer Today (Ferlay et al. 2020). For DLBCL, ENKTL-NT and BL, the proportion as part of a larger group of NHLs was determined before factoring EBV-related case proportion as determined through meta-analysis (Supplementary document). *EBV* Epstein–Barr virus, *NPC* nasopharyngeal carcinoma, *GC* gastric carcinoma, *HL* Hodgkin lymphoma, *BL* Burkitt Lymphoma, *DLBCL* diffuse large B-cell lymphoma, *ENKTL-NT* extranodal NK/T-cell lymphoma, nasal type

^1^de Martel et al. 2020

^2^Range of EBV-related cases as a percentage of global case range for the respective cancer(s)

^3^Haverkos et al. (2016) and Hue et al. 2020

## Supplementary Information

Below is the link to the electronic supplementary material.

**Supplementary Figure 1: Forest plots of pooled proportions of DLBCL cases out of NHL cases**. Subgroup analyses were performed according to the location of the study (East and South-East Asia, Latin America and the Rest of the world). (BMP 2389 kb)

**Supplementary Figure 2: Forest plots of pooled proportions of EBV-positive DLBCL cases out of DLBCL cases**. Subgroup analyses were performed according to the location of the study (East and South-East Asia, Latin America and the Rest of the world). Cut-off threshold for EBV positivity was restricted to 20% of more tumor cells staining positive for EBER. (BMP 2271 kb)

**Supplementary Figure 3: Forest plots of pooled proportions of ENKTL cases out of NHL cases**. Subgroup analyses were performed according to the location of the study (East and South-East Asia, Latin America and the Rest of the world). (BMP 2063 kb)

Supplementary file4 (DOCX 25 kb)

## Data Availability

Source data used during the current study are available from the Cancer Today website, (https://gco.iarc.fr/today/online-analysis-table). All datasets generated during the current study are available from the corresponding author on reasonable request.

## Abbreviations

ABVD: Adriamycin, bleomycin, vinblastine and dacarbazine
BL: Burkitt lymphoma
CAEBV: Chronic active EBV infection
CDKN2A: Cyclin-dependent kinase inhibitor 2A
CHOP: Cyclophosphamide, doxorubicin, vincristine and prednisone
CIN: Chromosomal instability
EBER: Epstein–Barr virus-encoded RNA
eBL: Endemic Burkitt lymphoma
EBV: Epstein–Barr virus
EBVaGC: Epstein–Barr virus-associated Gastric Carcinoma
EBVMCU: EBV-positive mucocutaneous ulcer
ENKTL-NT: Extranodal NK/T-cell lymphoma, nasal type
GC: Gastric carcinoma
GS: Genomically stable
HHV4: Human herpesvirus 4
HIV: Human immunodeficiency virus
HL: Hodgkin lymphoma
HLA: Human leukocyte antigen
HRS: Hodgkin and Reed–Sternberg
IARC: International Agency for Research on Cancer
IGH: Immunoglobulin heavy chain
IM: Infectious mononucleosis
ISH: In situ hybridization
LP: Lymphocyte-predominant
LPD: Lymphoproliferative disorder
MLH1: MutL homolog 1
MSI: Microsatellite instability
NHL: Non-Hodgkin lymphoma
NLPHL: Nodular lymphocyte-predominant Hodgkin lymphoma
NOS: Not otherwise specified
NPC: Nasopharyngeal carcinoma
PD-1: Programmed cell death protein 1
PD-L1: Programmed death-ligand 1
PFS: Progression-free survival
PTCL: Peripheral T-cell lymphoma
R-CHOP: Rituximab, cyclophosphamide, doxorubicin, vincristine and prednisone
SMILE: Steroid (dexamethasone), methotrexate, ifosfamide, l-asparaginase and etoposide
SNPs: Single nucleotide polymorphisms
STCLC: Systemic EBV-positive T-cell lymphoma of childhood
TCGA: The Cancer Genome Atlas Research Network
WHO: World Health Organization
