# Supplementary figures and images for: Estimating the global burden of Epstein–Barr virus-related cancers
Source: J Cancer Res Clin Oncol. 2021 Oct 27;148(1):31–46. doi: 10.1007/s00432-021-03824-y (PMC8752571; doi:10.1007/s00432-021-03824-y)

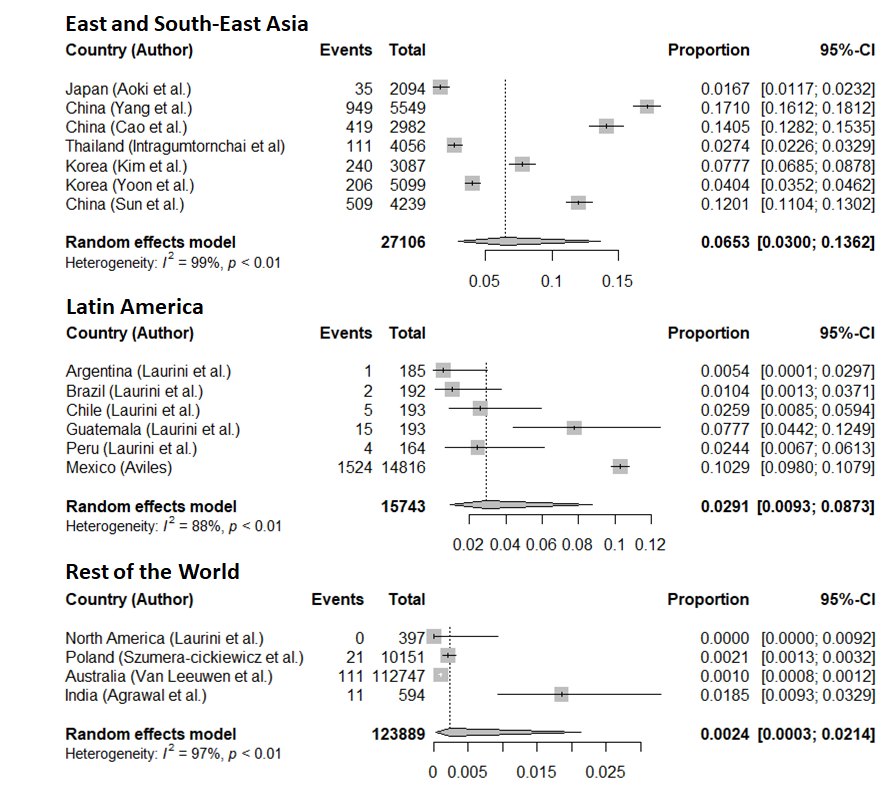

Supplement: Supplementary file 1 — Supplementary Figure 1: Forest plots of pooled proportions of DLBCL cases out of NHL cases. Subgroup analyses were performed according to the location of the study (East and South-East Asia, Latin America and the Rest of the world). (BMP 2389 kb) [file 432_2021_3824_MOESM1_ESM.bmp]

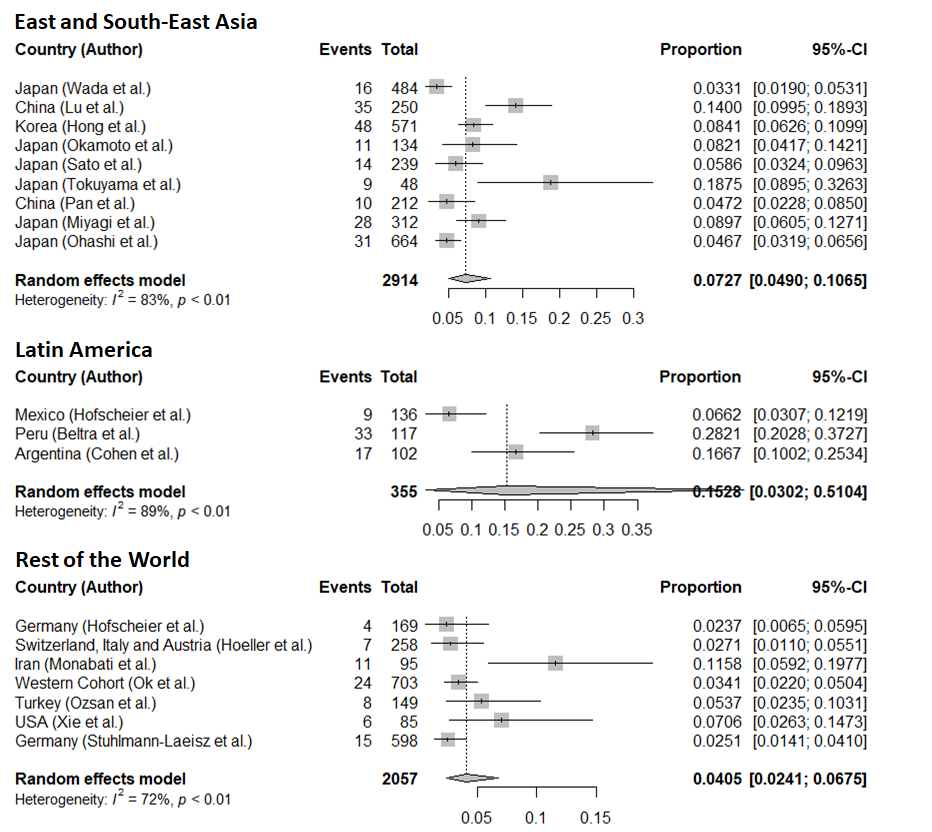

Supplement: Supplementary file 2 — Supplementary Figure 2: Forest plots of pooled proportions of EBV-positive DLBCL cases out of DLBCL cases. Subgroup analyses were performed according to the location of the study (East and South-East Asia, Latin America and the Rest of the world). Cut-off threshold for EBV positivity was restricted to 20% of more tumor cells staining positive for EBER. (BMP 2271 kb) [file 432_2021_3824_MOESM2_ESM.bmp]

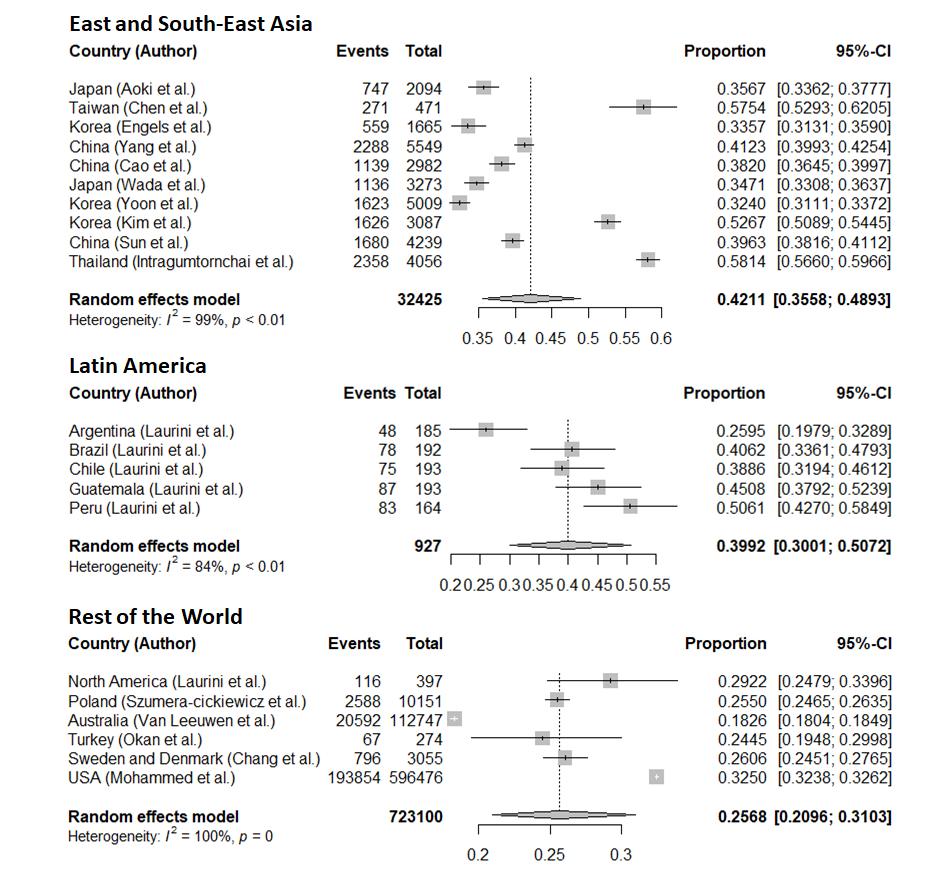

Supplement: Supplementary file 3 — Supplementary Figure 3: Forest plots of pooled proportions of ENKTL cases out of NHL cases. Subgroup analyses were performed according to the location of the study (East and South-East Asia, Latin America and the Rest of the world). (BMP 2063 kb) [file 432_2021_3824_MOESM3_ESM.bmp]
