# Supplementary material for: Estimating the global burden of Epstein–Barr virus-related cancers
Source: J Cancer Res Clin Oncol. 2021 Oct 27;148(1):31–46. doi: 10.1007/s00432-021-03824-y (PMC8752571; doi:10.1007/s00432-021-03824-y)
Supplement: Supplementary file 4 — Supplementary file4 (DOCX 25 kb) [file 432_2021_3824_MOESM4_ESM.docx]

**Supplementary Table 1: Regional EBV association, incidence and mortality of HL and GC**

| Hodgkin Lymphoma (HL) | | | | | |
| --- | --- | --- | --- | --- | --- |
| Region | % EBV-related (95% CI)^1^ | Incidence (95% CI)^2^ | EBV associated incidence (range^*^) | Mortality (95% CI)^2^ | EBV associated mortality (range^*^) |
| North America | 32 (25-39) | 9,077 (8,874-9,284) | 2,905 (2,219-3,621) | 1,064 (993-1,140) | 340 (248-445) |
| Latin America | 60 (54-67) | 10,634 (9,444-11,975) | 6,380 (5,099-8,023) | 2,835 (2,549-3,154) | 1,701 (1,376-2,113) |
| Africa | 74 (65-82) | 10,815 (7,994-14,632) | 8,003 (5,195-11,998) | 4,315 (3,060-6,086) | 3,193 (1,988-4,990) |
| Asia | 56 (52-60) | 31,742 (30,266-33,290) | 17,776 (15,738-19,974) | 11,079 (9,115-13,466) | 6,204 (4,739-8,080) |
| Europe | 36 (32-39) | 19,858 (18,735-21,049) | 7,149 (5,995-8,209) | 3,953 (3,533-4,423) | 1,423 (1,130-1,725) |
| Oceania | 29 (10-58) | 961 (870-1,062) | 279 (87-615) | 130 (95-178) | 38 (9-103) |
| Gastric Carcinoma (GC) | | | | | |
| North America | 12 (9-15)^3^ | 29,772 (29,398-30,151) | 3,424 (2,487-4,652) | 13,391 (13,128-13,658) | 1,540 (1,111-2,107) |
| Latin America |  | 67,617 (64,628-70,744) | 7,776 (5,468-10,916) | 53,392 (52,128-54,687) | 6,140 (4,410-8,438) |
| Africa | 9 (8-10)^4^ | 32,402 (26,783-39,200) | 2,819 (2,070-3,889) | 27,945 (22,502-34,705) | 2,431 (1,739-3,443) |
| Asia |  | 819,944 (805,161-834999) | 71,335 (62,239-82,832) | 575,206 (563,091-587,582) | 50,043 (43,527-58,288) |
| Europe |  | 136,038 (133,409-138,719) | 11,835 (10,313-13,761) | 96,997 (94,937-99,101) | 8,439 (7,339-9,831) |
| Oceania |  | 3,300 (3,144-3,527) | 287 (243-350) | 1,862 (1,715-2,022) | 162 (132-201) |

^1^Plummer et al. 2016.

^2^Ferlay et al. 2020 and Sung et al. 2021.

^3^Carrasco-Avino et al. 2017.

^4^Tavakoli et al. 2020.

^*^Estimated by multiplying the lower and upper limit of the 95% confidence intervals (CI) for EBV-related proportions with the lower and upper limit of the 95% confidence intervals for incidence or mortality respectively.

**Supplementary Table 2: Global EBV association, incidence and mortality of NPC and BL**

| Cancer | % EBV association^5^ | Incidence  (95% CI)^2^ | EBV associated incidence (range^#^) | Mortality  (95% CI)^2^ | EBV associated mortality (range^#^) |
| --- | --- | --- | --- | --- | --- |
| Nasopharyngeal Carcinoma (NPC) | 84 | 133,354  (124,737-142,566) | 112,838  (105,547-120,633) | 80,008  (72,848-87,871) | 67,699 (61,641-74,352) |
| Burkitt Lymphoma (BL) | 55 | 12,000^5^ | 6,600^5^ | 5,727  (5,609-5,8347)^6^ | 3,150 (3,085-3,216)^6^ |

^2^Ferlay et al. 2020 and Sung et al. 2021.

^5^de Martel et al. 2020.

^6^Estimated by calculating the proportion of BL incidence out of NHL incidence and multiplying by mortality. NHL incidence and mortality were retrieved from the GLOBOCAN database from the Cancer Today website (Ferlay et al. 2020; Sung et al. 2021).

^#^Estimated by multiplying EBV-associated proportions with the lower and upper limit of the 95% CI for incidence or mortality respectively.

**Supplementary Table 3:**

| Diffuse Large B-cell Lymphoma (DLBCL) | | | | | | |
| --- | --- | --- | --- | --- | --- | --- |
| Region | % Proportion DLBCL of NHL (95% CI)^7^ | Incidence (range^^^) | Mortality (range^^^) | % EBV-related (95% CI)^7^ | EBV-related incidence (range^*^) | EBV-related mortality (range^*^) |
| Eastern and South-Eastern Asia | 42 (36-49) | 72,592 (60,244-85,915) | 39,925 (33,041-47,386) | 7 (5-11) | 5,277  (2,952-9,150) | 2,902 (1,619-5,047) |
| Latin America | 40 (30-51) | 15,923 (11,327-21,378) | 7,646 (5,524-10,109) | 15 (3-15) | 2,433  (342-10,911) | 1,168  (167-5,160) |
| Rest of the world | 26 (21-31) | 85,278 (68,951-103,971) | 37,448 (30,011-46,071) | 4 (2-7) | 3,454  (1,662-7,018) | 1,517  (723-3,110) |
| Extranodal NK/T-Cell Lymphoma, Nasal Type (ENKTL-NT) | | | | | | |
| Eastern and South-Eastern Asia | 7 (3-14) | 11,257 (5,080-23,915) | 6,191 (2,786-13,190) | 100 | 11,257 (5,080-23,915) | 6,191 (2,786-13,190) |
| Latin America | 3 (0.9-9) | 1,161 (351-3,680) | 557 (171-1,740) |  | 1,161 (351-3,680) | 557 (171-1,740) |
| Rest of the world | 0.2 (0.03-2) | 797 (99-7,170) | 350 (43-3,177) |  | 797 (99-7,170) | 350 (43-3,177) |

^7^Estimated through meta-analyses described in text and in the figures below.

^^^Estimated by multiplying the lower and upper limit of the 95% CI for DLBCL or ENKTL-NT proportions of NHL with the respective lower and upper limit of the 95% CI for regional NHL incidence or mortality retrieved from the GLOBOCAN database from the Cancer Today website (Ferlay et al. 2020; Sung et al. 2021).

^*^Estimated by multiplying the lower and upper limit of the 95% CI for EBV-related proportions with the lower and upper range of DLBCL or ENKTL-NT incidence or mortality (estimated previously^^^) respectively.

**References**

Carrasco-Avino G, Riquelme I, Padilla O, Villaseca M, Aguayo FR, Corvalan AH (2017). The conundrum of the Epstein-Barr virus-associated gastric carcinoma in the Americas. Oncotarget 8(43): 75687-75698.

de Martel C, Georges D, Bray F, Ferlay J, Clifford GM (2020). Global burden of cancer attributable to infections in 2018: a worldwide incidence analysis. Lancet Glob Health 8(2): e180-e190.

Ferlay J, Ervik M, Lam F, M. C, Mery L, Piñeros M, Znaor A, Soerjomataram I, Bray F. (2020). Global Cancer Observatory: Cancer Today. <https://gco.iarc.fr/today>. Retrieved 01 July, 2021.

Plummer M, de Martel C, Vignat J, Ferlay J, Bray F, Franceschi S (2016). Global burden of cancers attributable to infections in 2012: a synthetic analysis. Lancet Glob Health 4(9): e609-616.

Sung H, Ferlay J, Siegel RL, Laversanne M, Soerjomataram I, Jemal A, Bray F (2021). Global cancer statistics 2020: GLOBOCAN estimates of incidence and mortality worldwide for 36 cancers in 185 countries. CA Cancer J Clin.

Tavakoli A, Monavari SH, Solaymani Mohammadi F, Kiani SJ, Armat S, Farahmand M (2020). Association between Epstein-Barr virus infection and gastric cancer: a systematic review and meta-analysis. BMC Cancer 20(1): 493.
